# Ulnar nerve palsy after open carpal tunnel decompression: Case report and review of the literature

**DOI:** 10.4103/0970-0358.41117

**Published:** 2008

**Authors:** P. Yoong, A. Fattah, A. S. Flemming

**Affiliations:** St Andrews Centre for Plastic Surgery and Burns, Broomfield Hospital, Court Road, Chelmsford, Essex CM1 7ET UK

**Keywords:** Carpal tunnel, complications, nerve injury, ulnar nerve

## Abstract

Open carpal tunnel release is the commonest surgical treatment of median nerve compression at the wrist. Although successful in most cases, there are well described complications. We report a case of laceration of the deep motor branch of the ulnar nerve at the level of the hook of hamate following a complicated carpal tunnel decompression. Good surgical technique and knowledge of wrist anatomy are essential for performing this apparently simple procedure safely.

## INTRODUCTION

Carpal tunnel syndrome is the commonest nerve entrapment of the upper limb, with an estimated lifetime risk of 10%.[1] Surgical decompression of the transverse carpal ligament has proven value, producing significant relief of pain and paraesthesia in more than 70% of patients.[2]

Complications are well described. MacDonald *et al.*, describe an incidence of 12% following 186 cases of open carpal tunnel release.[3] One can divide these into persistence of preoperative symptoms and new symptoms. The former is most often due to incomplete release of the flexor retinaculum. New postoperative symptoms are often iatrogenic, associated with damage to branches of the median nerve, most commonly the palmar cutaneous branch, leading to painful neuroma formation, a painful scar or altered sensibility. Injury to the ulnar nerve is a less common complication of carpal tunnel decompression. We report a case of complete laceration to the deep motor branch following an apparently routine carpal tunnel decompression.

A 76-year-old woman was referred with a 4 month history of nocturnal paraesthesia and numbness of both hands in the median nerve distribution, consistent with bilateral carpal tunnel syndrome. Nerve conduction studies showed severe compression of the median nerve on the right and mild compression on the left. She was being treated for hypertension. A right carpal tunnel decompression was performed under local anaesthesia using an incision in line with the middle of the fourth ray. Persistent bleeding, which required deflation and re-inflation of the tourniquet on four occasions during surgery, complicated the operation making dissection difficult. Despite this the procedure was apparently performed without any adverse effects.

She recovered from surgery well, but complained of weakness of grip and loss of pinch power on follow-up at 2 weeks and at 3 months. It was thought that this was a transient ulnar neuropathy. However, 8 months following her surgery there was obvious wasting of the hypothenar eminence with clawing of the little and ring fingers and decreased sensation in the ring finger. She was initially treated with regular physiotherapy and anti-clawing splints. Repeat nerve conduction studies showed a lesion of the deep motor branch of the right ulnar nerve that was not present preoperatively.

**Figure 1:**
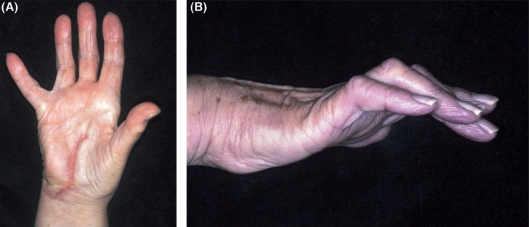
(A and B) Right hand 13 months after initial surgery and 1 month after repair of deep motor branch of ulnar nerve. There is visible clawing of the little and ring fingers, a positive Wartenberg's sign and wasting of the hypothenar eminence

When the palm was explored at 12 months, we found that the deep branch of the ulnar nerve had been divided at the base of the hook of hamate with extensive scarring and neuroma formation. The carpal tunnel was reopened, the neuroma excised and microneural repair performed by direct coaptation. In clinic 3 months later, there was little objective improvement in the function of her right ulnar nerve [Figure 1(A and B)].

## DISCUSSION

The anatomy of the ulnar nerve is well described. Passing down the forearm beneath flexor carpi ulnaris, a palmar cutaneous branch emerges from 5 cm proximal to the wrist to supply skin over the mid-palm. A dorsal sensory branch emerges from its medial border around 5 cm proximal to the pisiform. The ulnar nerve continues distally through the Guyon′s canal between the pisiform and the hook of hamate. At this point there is a bifurcation into a superficial sensory and deep motor branch. The latter arises from the ulnar side of the nerve, passes beneath the main trunk and is joined by the deep branch of the ulnar artery to enter the deep part of the palm adjacent and just distal to the hook of hamate, to supply most of the small muscles of the hand[4]

Ulnar nerve palsy is a rare complication of open carpal tunnel decompression. There are four previously reported cases of injury to the deep motor branch. Favero[5] described the first case in 1987. Terrono *et al.*,[6] reported 3 more cases. Two of the incisions used were described as “in line with the ring finger”^6^, one as being “1 cm ulnar and parallel to the thenar crease”^6^ and one as being “1 cm ulnar to the centre of the palm”^5^. Microsurgical repair was performed in all four patients, with some subsequent improvement in hand function.

A variety of incisions have been described over the years, to reduce the risk of damage to the palmar cutaneous branches of the median nerve (PCBMN) or ulnar nerve (PCBUN) nerves, as this is believed to have a role in postoperative scar discomfort.[7]

The classical incision for open carpal tunnel release is in line with the radial border of the ring finger. In 1973, Taleisnik[8] proposed an incision to the ulnar side of the fourth ray to reduce the risk of damage to the PCBMN, but Matloub *et al.*,[9] suggest that it may increase the risk of damage to the PCBUN as the distribution of the PCBUN is much more variable. Engber and Gmeiner[10] found that the branches of the PCBMN extended radially towards the fourth ray axis but did not cross it, but Born and Mahoney[11] report that branches of the PCBUN cross the carpal tunnel incision in line with the fourth ray in 12.5% of cadaver palms. Martin *et al.*,[12] performed a dissection of the ulnar and median nerves in 25 cadaveric hands, concluding that there is no true inter-nervous plane through which to approach the carpal canal. Thus, although entering the ulnar part of the carpal canal may reduce both the risk of scarring over the median nerve and damage to the PCBMN, it may increase the risk of injury to the branches of the ulnar nerve.

Nevertheless, cases of damage to the motor branch of the ulnar nerve during carpal tunnel release are rare. In our patient, it is probable that a poorly inflated tourniquet or high blood pressure led to bleeding and poor operating conditions. In such cases the surgeon should either abandon the procedure or use basic surgical principles to prevent problems. Although considered to be “the simplest of operations” complications still occur and meticulous surgical technique and careful dissection are prerequisite to avoiding complications.

## CONCLUSION

Though rare, ulnar nerve injury should be considered in cases with hypothenar and interosseous muscle weakness, ulnar nerve sensory disturbance and poor hand function following carpal tunnel release.
